# Assessing the role and impact of research in clinical practice among acupuncturists in western countries: a multinational cross-sectional survey

**DOI:** 10.3389/fmed.2024.1331184

**Published:** 2024-07-31

**Authors:** Matthias Huemer, Sandro Graca, Sarah Bitsche, Guenter Hofmann, Mike Armour, Martin Pichler

**Affiliations:** ^1^Department of Internal Medicine, Division of Oncology, Palliative Care Unit, Medical University of Graz, Graz, Austria; ^2^Northern College of Acupuncture, York, United Kingdom; ^3^School of Health and Society, Faculty of Education, Health and Wellbeing, University of Wolverhampton, Wolverhampton, United Kingdom; ^4^NICM Health Research Institute, Western Sydney University, Penrith, NSW, Australia; ^5^Translational Health Research Institute, Western Sydney University, Penrith, NSW, Australia; ^6^Medical Research Institute of New Zealand (MRINZ), Wellington, New Zealand; ^7^Translational Oncology, University Hospital of Augsburg, Augsburg, Germany

**Keywords:** evidence-informed practice, acupuncture, educational programs, public health, continuing professional development

## Abstract

**Background:**

Evidence-informed practice is crucial to perform safe and efficient health interventions. In recent years, the evidence base of acupuncture continuously increased leading to the integration of acupuncture into clinical guidelines by various leading medical associations worldwide. At the same time, recent studies showed that licensed acupuncturists are rarely utilizing scientific research to inform their practice.

**Methods:**

This descriptive study using an online survey assessed the role of evidence-informed practice of acupuncturists in Austria, Germany, the United States of America, Australia, and New Zealand and aimed to determine critical factors relevant for promoting research literacy including demographical data, data about the clinical practice patterns, and the role and value of different information sources of traditional, complementary and integrative medicine (TCIM) practitioners.

**Results:**

In total, 404 acupuncturists completed the online survey that included questions about demographic characteristics, the role and value of research in clinical practice, and details about the amount and type of continuing professional education. Univariate and multivariate analysis was used to determine significant predictors of the outcome variable “importance of research in clinical practice” (numerical rating scale, 0 to 100). The results showed that the majority of acupuncturists use certified courses as primary source of continuing professional education and value experts’ opinions as the most reliable source of information. Multivariate analysis showed that the importance of research is dependent on the interest in research, an interdisciplinary learning environment, and positive experiences with research including if an acupuncture study ever changed the clinical practice of practitioners.

**Conclusion:**

Future educational programs should therefore focus on an interactive format aiming to promote skills to critically assess the value and practical use of research studies to improve the general practice of acupuncture.

## 1 Introduction

Evidence-informed practice is a fundamental principle in modern evidence-based medicine. It assures best clinical practice by balancing highest effectiveness and patient safety (1). To fulfill this, practitioners need the skills to critical evaluate and interpret research before translating it into clinical practice (2). However, one of the greatest barriers for the implementation of evidence-based practice in medicine, regardless of the medical discipline, is research literacy, the capability of health care personnel to critical appraise and interpret clinical research (3). Additionally, secondary factors including education level, time-issues, work-life balance, and accessibility of research articles are barriers to ongoing education and incorporation of research findings in their clinical practice (4–7).

In recent years, there has been a substantial amount of research on acupuncture and a focus on improving the evidence base for various conditions (8, 9). Currently, major medical associations suggest acupuncture for the treatment of various conditions, especially for the treatment of chronic pain (10, 11) and also pain management in oncology (12), which led to a change of health care policies promoting insurance coverage of acupuncture (12–18). Therefore, acupuncturists are hired increasingly by medical facilities to provide an overall integrative treatment in the United States of America (19). However, acupuncture is mostly taught and performed based on traditional medical systems rather than current scientific evidence according to a recent systematic reviews and surveys among acupuncturists in Norway, the United States of America, Australia and New Zealand (6, 13, 20, 21). This limits acupuncturists in reading and interpreting acupuncture research as most studies lack some key aspects of traditional theoretical frameworks, making them hard to reproduce for an acupuncturist trained only in traditional medical systems (22). A multitude of additional factors were reported by acupuncturists as reasons for not utilizing acupuncture research for their clinical practice, including time constraints and also not valuing peer-reviewed publications as the best source of information to inform clinical practice (6, 21).

Different strategies for promoting research literacy among healthcare personnel exist and show promising results including workshops, journal clubs, research fellowships, and formal university courses of either interactive and student-centered or teacher-centered formats. Among all educational strategies, an interactive and student-centered approach showed the best results for improving research literacy (3). However, any educational program will fail if healthcare personnel, including acupuncturists, are not willing to participate. Enhancing the understanding of relevance and value of research findings is the first step toward improving research literacy, as it can motivate practitioners to seek further education about research interpretation and external validity for use in clinical practice.

Currently, factors associated to the attitude of acupuncturists toward research are poorly understood. This multinational survey among licensed acupuncturists was designed to assess the importance of research for clinical practice, as well as possible influencing factors to improve our understanding of how practitioners may be motivated toward educational programs designed to improve research literacy.

## 2 Methods

### 2.1 Study design

We designed a descriptive study using an online survey. The primary outcome was to assess the importance and value of research in clinical practice of acupuncture. Therefore, an anonymous self-completion questionnaire was designed to collect data about the participating acupuncturists’ demographics, annual hours spent in continuing professional development (CPD), and both primary and most trusted source of information for their clinical practice. The study was approved by the local ethics committee (Ethics Committee of the Medical University of Graz, Austria; document number 34-432 ex 21/22).

### 2.2 Survey questionnaire and outcome measurement

For the primary outcome, we asked the respondents to rate the importance of research on a 0 to 100 numerical rating scale, where higher scores reflected greater importance of research for clinical practice. The questionnaire included open and closed questions in single-choice categorical response format. Demographical data describing the diversity of the participants included profession, age, place of living, education level, education in acupuncture, place of practice, experience in years, and average patient volume per week. Additional questions about the value of research in clinical practice included general interest in research, if a study ever changed their clinical practice, participation in acupuncture research, and number of research papers read per year. Participants were also asked if the principles of traditional Chinese medicine (TCM) remain valid in the context of modern medicine and if these can be replicated in a research study. The survey questionnaire was primarily designed by M.H. and S.G. using previous published surveys assessing the research literacy among acupuncturists (4, 6, 13, 23, 24). The final version of the questionnaire was reviewed by M.A. and M.P. Each survey item and its response categories are listed in Table 1.

**Table 1:** Demographic data and univariable analysis of “Importance of Research” (Total = 404).

	*N*	%	Mean	SD	*p*-value
Profession	399				0.79
Acupuncturist	180	45.1%	58.6	24.6	
Acupuncturist and Herbalist	219	54.9%	59.4	26.1	
Missing	5	1.2%			
Medical doctor	393				0.96
Yes	107	27.2%	59.6	28.1	
No	286	72.8%	59.4	24.5	
Missing	11	2.7%			
Age	400				0.07
18–35	35	8.8%	74.3	24.5	Ref.
36–45	82	20.5%	59.4	23.1	0.08
46–55	149	37.2%	57.3	24.4	0.02
56–65	93	23.2%	58.5	26.1	0.04
>65	41	10.2%	57.1	30.6	0.06
Missing	4	1.0%			
Country	364				<0.01
Austria	78	21.4%	59.5	27.5	Ref.
United States of America	118	32.4%	66.2	20.0	0.33
Germany	106	29.1%	48.4	26.2	0.04
Australasia	62	17.0%	62.6	24.3	1.00
Missing	40	9.9%			
Highest education level	392				<0.01
High school	23	5.9%	45.4	20.5	Ref.
College	50	12.8%	49.6	26.7	1.00
University	319	81.4%	61.3	25.2	0.02
Missing	12	3.0%			
Highest education level in acupuncture	389				<0.01
Short-term course (< 100 h)	19	4.9%	56.5	31.9	Ref.
Diploma or > 100 h	201	51.7%	54.1	26.1	1.00
Master’s degree	129	33.2%	61.8	24.0	1.00
PhD	40	10.3%	70.0	20.3	0.41
Missing	15	3.7%			
Hours spent in continuing education per year	395				0.66
<10	9	2.3%	50.0	29.6	Ref.
10–50	265	67.1%	59.3	24.4	0.76
51–100	121	30.6%	59.7	27.8	0.74
Missing	9	2.2%			
Place of practice	356				<0.01
My own solo clinic	258	72.5%	54.4	25.0	Ref.
A hospital	33	9.3%	75.8	22.9	<0.01
A multidisciplinary clinic	65	18.3%	69.6	22.7	<0.01
Missing	39	9.7%			
Do you think that the principles and concepts of TCM remain valid in the context of modern medical practice?	318				0.47
Yes	300	94.3%	59.2	25.4	
No	18	5.7%	64.5	28.9	
Missing	86	21.3%			
Do you think that the principles of a TCM treatment can be replicated in a research study?	314				0.04
Yes	245	78.0%	61.5	24.5	
No	69	22.0%	53.6	28.0	
Missing	90	22.3%			
Interested in research	319				<0.01
No	11	3.4%	22.1	22.4	
Yes	308	96.6%	60.7	24.5	
Missing	85	21.0%			
Participation in acupuncture research	318				<0.01
Yes	78	24.5%	68.8	23.8	
No	240	75.5%	55.3	25.2	
Missing	86	21.3%			
Source of information	323				<0.01
Online literature database search (e.g., PubMed, Google Scholar, etc.)	89	27.6%	68.9	20.5	Ref.
Conferences	29	9.0%	69.1	24.1	1.00
Courses or continuing professional development (CPD/CEU)	127	39.3%	50.5	24.5	<0.01
Print media (Journals, books, etc.)	35	10.8%	58.2	30.5	0.14
Webinars or Seminars by experts	43	13.3%	55.9	25.6	0.02
Missing	81	20.1%			
Did an acupuncture study ever change your clinical practice?	317				<0.01
No	107	33.8%	44.5	25.2	
Yes	210	66.2%	66.3	22.5	
Missing	87	21.5%			
Most reliable source of information	318				<0.01
Research papers	113	35.5%	69.3	24.6	Ref.
Experts	137	43.1%	52.8	23.5	<0.01
Books	27	8.5%	58.2	25.6	0.15
Conferences	41	12.9%	52.4	26.9	<0.01
Missing	86	21.3%			
Number of papers read per year	323				<0.01
< 10	113	35.0%	48.2	26.1	Ref.
10–20	115	35.6%	58.0	23.7	<0.01
21–40	50	15.5%	69.0	20.0	<0.01
>40	45	13.9%	78.2	19.0	<0.01
Missing	81	20.1%			

#### 2.2.1 Translation and piloting the questionnaire

The questionnaire was available in two languages, English and German. The first version was created in English and then translated to German by three native speakers (M.H. German, S.G. English, and external professional translator) using the forward-backward-translation process. After reviewing the final two versions, both versions were piloted by 10 German or English-speaking acupuncturists asking them to rate the understandability, readability, and user-friendliness of the questionnaire on a scale of one to 10 with higher scores indicating a better understandability, readability, or user-friendliness. Additionally, we offered the piloting acupuncturists a free-text field to provide additional suggestions on how to improve the questionnaire. The questionnaire showed good understandability and readability, requiring only minor orthographical and grammatical adaptions.

### 2.3 Participants

Acupuncturists were invited to participate in this study if they were 18 years or older, held a current license for acupuncture practice, and were currently living in Austria, Germany, the United States of America, Australia, and New Zealand.

### 2.4 Recruitment strategies

Professional organizations representing acupuncture and Chinese medicine were asked to distribute the survey-link on their social media channels and via email between October 2022 and December 2022. The survey was open from October 1st, 2022, to January 15th, 2023. The participating organizations are listed in Table 2. Before starting the survey, participants were asked to give their consent to participate and agree to the use of their provided data using the built-in e-consent tool of the Research Electronic Data Capture (REDCap) system (25, 26). The survey participants were not offered any type of incentives as a means to increase their willingness to participate or for compensation.

**Table 2:** Participating organizations.

Organization name	Abbreviation	Country
Wiener Schule für Traditionelle Chinesische Medizin	WSTCM	Austria
Österreichische Gesellschaft für kontrollierte Akupunktur	OGKA	Austria
Societas Medicinae Sinesis	SMS	Germany
Arbeitsgemeinschaft für Klassische Akupunktur und Traditionelle Chinesische Medizin	AGTCM	Germany
Hospital Handbook Project	HHP	USA
American Society of Acupuncturists	ASA	USA
National Certification Commission for Acupuncture and Oriental Medicine	NCCAOM	USA
Australian Acupuncture and Chinese Medicine Association	AACMA	Australia
Acupuncture New Zealand	Acupuncture NZ	New Zealand

USA, United States of America.

### 2.5 Data management and analysis

The survey data was collected and managed using the REDCap online survey and distribution tool hosted by the Medical University of Graz, Austria (25, 26). We then exported the data from the REDCap system to RStudio (“Ghost Orchid” Release, R version 4.0.3) (27). All variables with less than 30% missing data were included, and missing data was reported. Descriptive statistics included means, standard deviations (SD), or numbers and percentages. For univariable analysis of two groups, we used Student’s *t*-test or Fisher’s exact test, for more than two groups we used analysis of variance (ANOVA), Kruskal-Wallis-Test, or linear regression. The significance level was set at *p* < 0.05 and corrected using the Bonferroni correction in multiple testing. For multivariable analysis, we built a linear regression model using the importance of research rating as the outcome variable and all variables with a *p*-value of <0.1 in the univariable analysis as predictors. The final number of predictor variables for the regression model was based on an exhaustive search algorithm selecting the variables yielding the highest adjusted R-square value (28). We did not use imputation methods to replace missing data. All analyses and plotting were performed using R version 4.0.3 and the package “ggplot2” (27, 29).

## 3 Results

### 3.1 Demographics

In total, 43,695 acupuncturists were invited by the participating professional organizations, of which 742 respondents completed the questionnaire (response rate 1.7%). For further analysis, 404 questionnaires fulfilled the predefined criteria of less than 30% missing data and current place of living. The majority of respondents were not medical doctors (72.8%), had a license in acupuncture and herbal medicine (54.9%), were 46–55 years old (37.2%), completed an education on university level (81.4%), and a diploma or > 100 h course in acupuncture (51.7%). The responding acupuncturists had a mean clinical experience of 15.1 years (standard deviation [SD] 9.8) and a mean patient volume of 34.9 (SD 50.0) per week. The majority (72.5%) of acupuncturists work in their own solo clinic. Respondents were distributed evenly among countries with the highest proportion in the United States of America (32.4%). The demographical data and univariable analysis are summarized in Tables 1, 3.

**Table 3:** Continuous variables from demographical data (Total = 404).

	*N* (%)	Mean	SD	*p*-value
Importance of research rating (NRS 0–100)	297 (73.5%)	59.3	25.5	
Clinical experience in years	357 (88.4%)	15.1	9.8	0.03
Average patient volume per week	348 (86.1%)	34.9	50.0	0.42

Demographical data and univariate analysis results of “Importance of research rating” (NRS, range 0–100). SD, standard deviation; TCM, traditional Chinese medicine; Ref., reference.

### 3.2 Role of research in clinical practice

The mean of the importance of research in clinical practice rating was 59.3 (SD 25.5) in the total population (Table 3). Almost all respondents (96.6%) stated to be interested in acupuncture research but a lower proportion (66.2%) stated that research ever changed their clinical practice. Additionally, only 24.5% ever participated actively in acupuncture research. Most acupuncturists (35.6%) read 10–20 research papers per year but almost the same proportion (35.0%) read less than 10 papers per year. Overall, 94.3% of the responding acupuncturists think that the principles of TCM remain valid in the context of modern medical practice and 78.0% think that it is possible to replicate those principles in a research study (Table 2).

### 3.3 Informing clinical practice

To further educate themselves, acupuncturists primarily participate in certified courses (39.3%) and think experts are the most reliable source of information (43.1%). Performing a literature search in online databases like Pubmed or Google Scholar is the second most utilized way to gain knowledge (27.6%) and 35.5% also think that research papers are the most important source. Regarding time commitment, 67.1% spend 10–50 h per year for continuing education (Table 2).

### 3.4 Univariable analysis

Univariable analysis was performed using the importance of research rating as the outcome variable. Age (ANOVA, *p* = 0.07), country (ANOVA, *p* < 0.01), highest education level (ANOVA, *p* < 0.01), highest education level in acupuncture (ANOVA, *p* < 0.01), place of practice (ANOVA, *p* < 0.01), if TCM can be replicated in a research study (*t*-test, *p* = 0.04), interest in research (*t*-test, *p* < 0.01), participation in acupuncture research (*t*-test, *p* < 0.01), source of information (ANOVA, *p* < 0.01), if an acupuncture study ever changed the clinical practice of the acupuncturists (*t*-test, *p* < 0.01), most reliable source of information (ANOVA, *p* < 0.01), number of papers read per year (ANOVA, *p* < 0.01), and clinical experience in years (linear regression, *ß* = −0.32, *p* = 0.03) showed significant differences in how participants rated the importance of research for clinical practice (Table 2).

**Figure 1:**
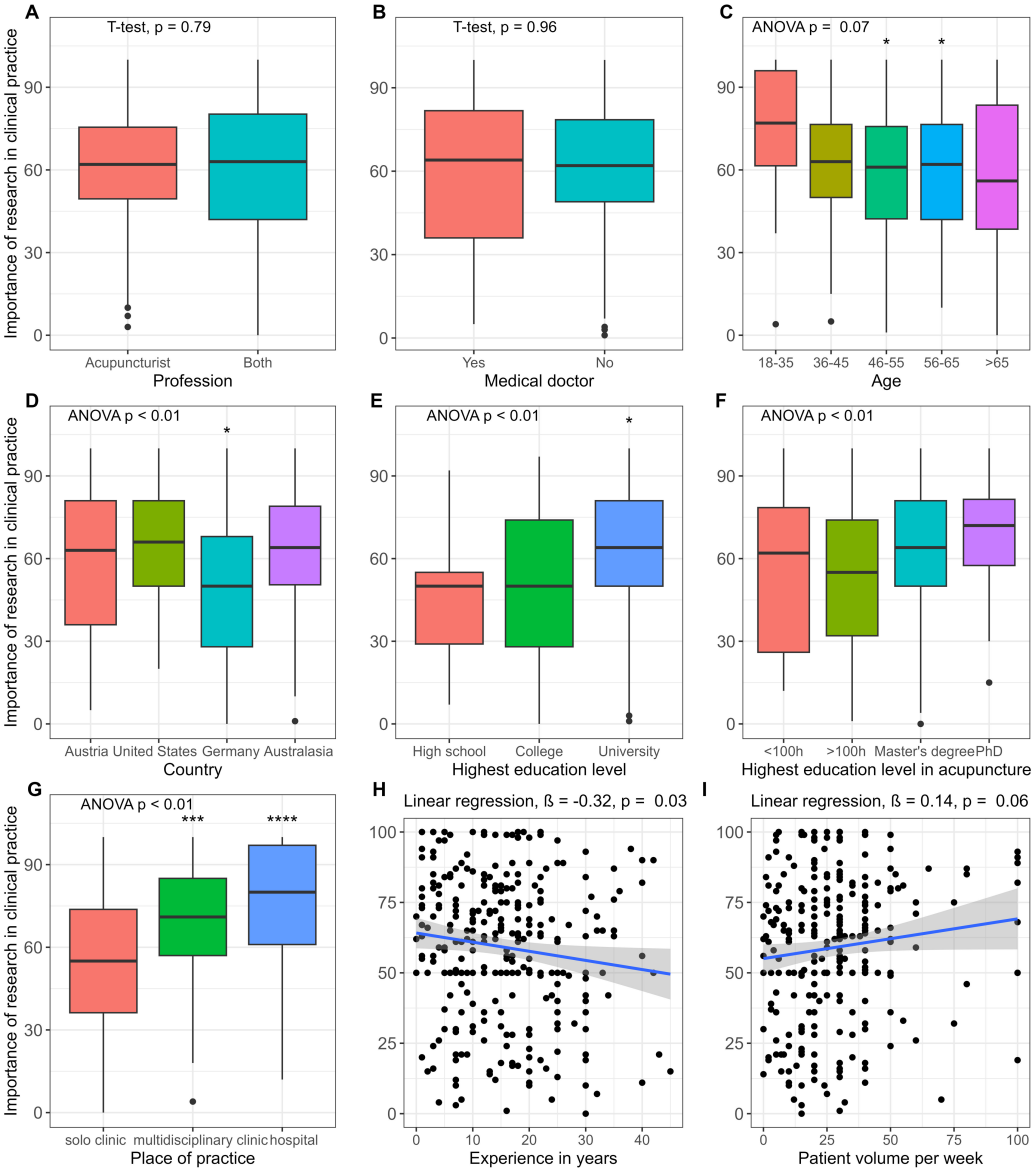
Univariable analysis of the importance of research in clinical practice by demographical data. **(A)** Profession, **(B)** Medical doctor, **(C)** Age, **(D)** Country, **(E)** Highest education level, **(F)** Highest education level in acupuncture, **(G)** Place of practice, **(H)** Experience in years, **(I)** Patient volume per week.

Additionally, variables with more than two outcome possibilities, a pairwise t-test was performed to determine factors that are significantly different to the reference group (first response category). All *p*-values were adjusted by Bonferroni correction for multiple testing. Statistical differences were found in the demographical data between age “18–35” (*M* = 74.3, SD = 24.5) and age “46–55” (*M* = 57.3, SD = 24.4, *p* = 0.02) and “56–65” (*M* = 58.5, SD = 26.1, *p* = 0.04), the country “Austria” (*M* = 59.5, SD = 27.5) and “Germany” (*M* = 48.4, SD = 26.2, *p* = 0.04), the highest education level “High school” (*M* = 45.4, SD = 20.5) and “University” (*M* = 61.3, SD = 25.2, *p* = 0.02), and place of practice “my own solo clinic” (*M* = 54.4, SD = 25.0) and “multidisciplinary clinic” (*M* = 69.6, SD = 22.7, *p* < 0.01) and “hospital” (*M* = 75.8, SD = 22.9, *p* < 0.01) (Figure 1).

**Figure 2:**
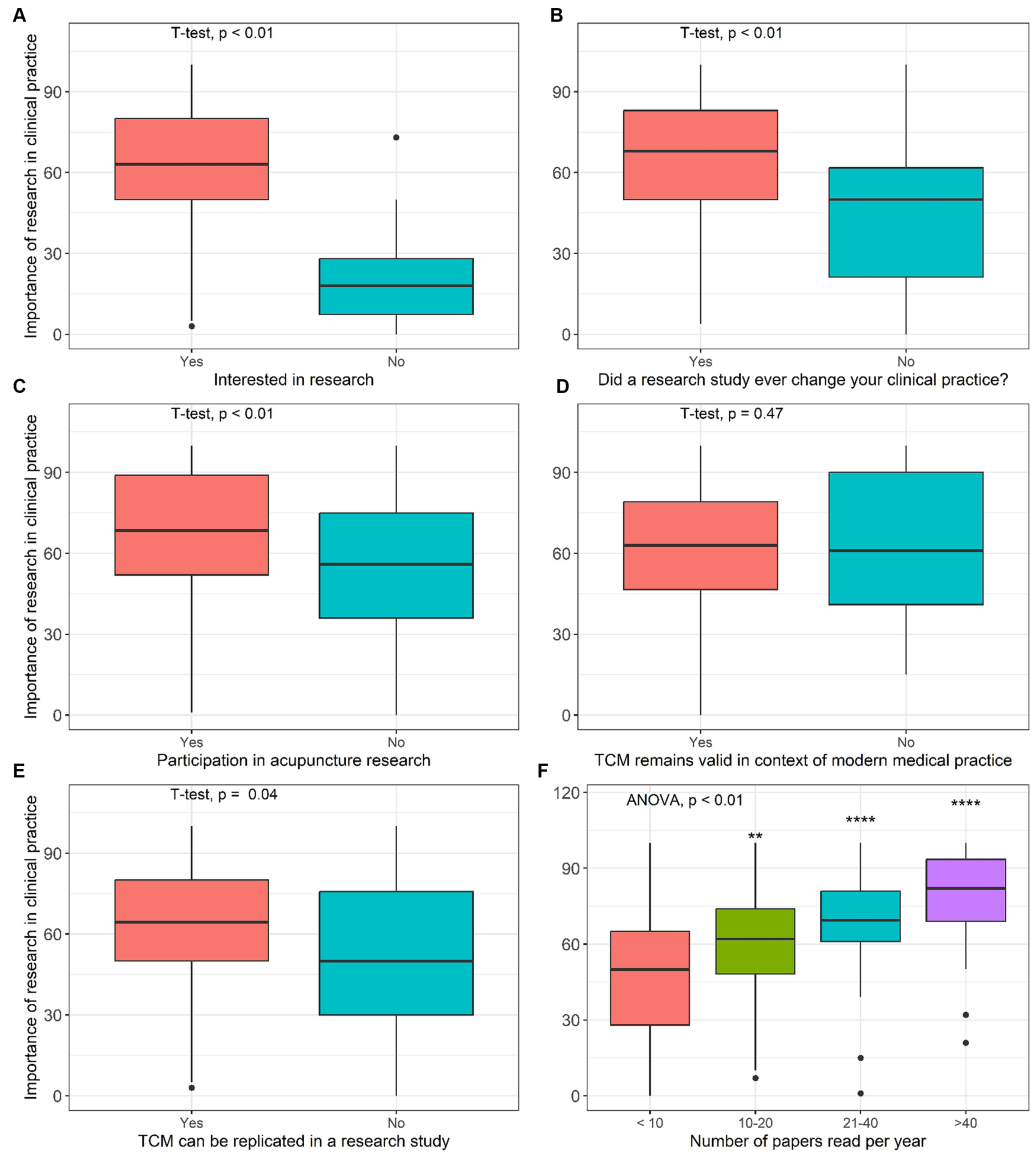
Univariable analysis of the importance of research in clinical practice by variables describing the value research. **(A)** Interested in research, **(B)** Dida research study ever change your clinical practice? **(C)** Participation in acupuncture research, **(D)** TCM remains valid in context of modern medical practice, **(E)** TCM can be replicated in a research study, **(F)** Number of papers read per year.

Additionally, numbers of papers read per year “<10” (*M* = 48.2, SD = 26.1) was significantly different to “10–20” (*M* = 58.0, SD = 23.7, *p* < 0.01), “21–40” (*M* = 69.0, SD = 20.0, *p* < 0.01), and “>40” (*M* = 78.2, SD = 19.0, *p* < 0.01) (Figure 2).

**Figure 3:**
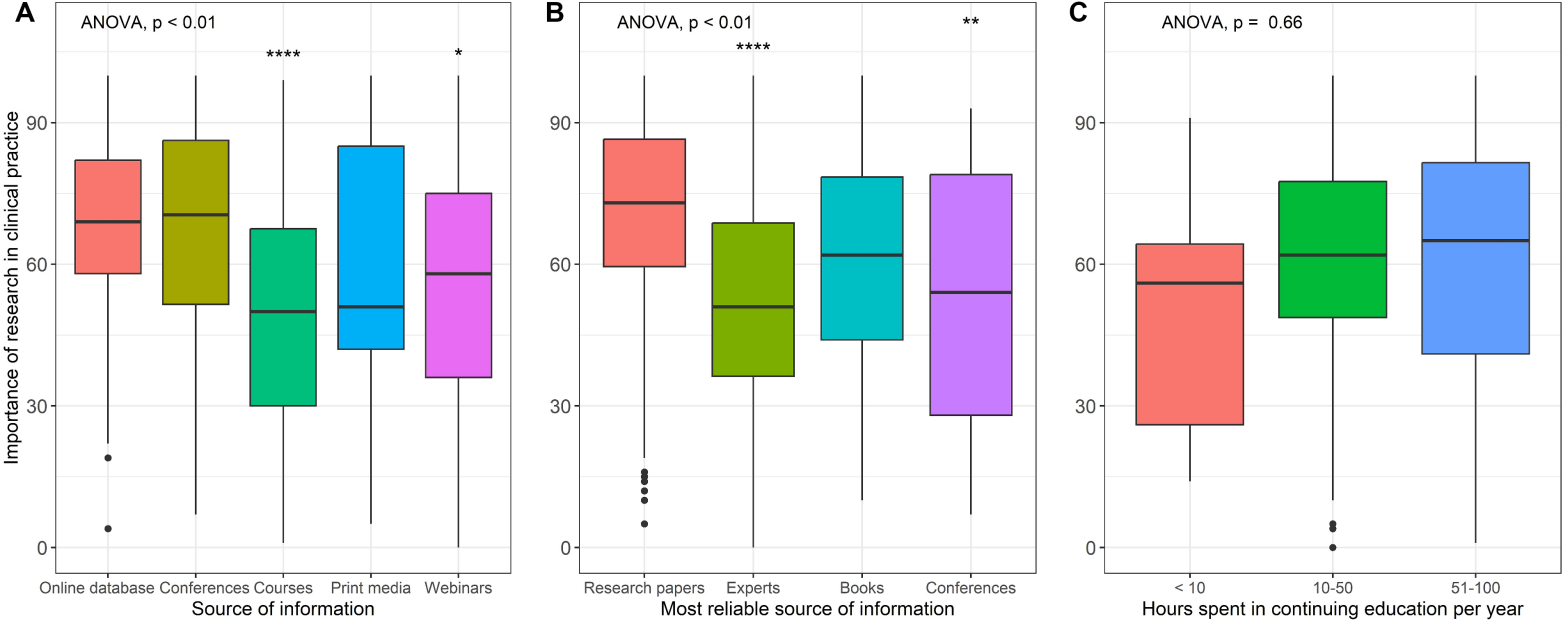
Univariable analysis of the importance of research in clinical practice by variables describing how practitioners inform their clinical practice. **(A)** Source of information, **(B)** Most reliable source of information, **(C)** Hours spent in continuing education per year.

Finally, statistical differences were also found between the source of information “online database” (MD = 68.9, SD = 20.5) compared to “courses” (MD = 50.5, SD = 24.5, *p* < 0.01) and “webinars” (MD = 55.9, SD = 25.6, *p* = 0.02), and the most reliable source of information being “research papers” (MD = 69.3, SD = 24.6) compared to “experts” (MD = 52.8, SD = 23.5, *p* < 0.01) and “conferences” (MD = 52.4, SD = 26.9, *p* < 0.01) (Figure 3).

### 3.5 Multivariable analysis

**Figure 4:**
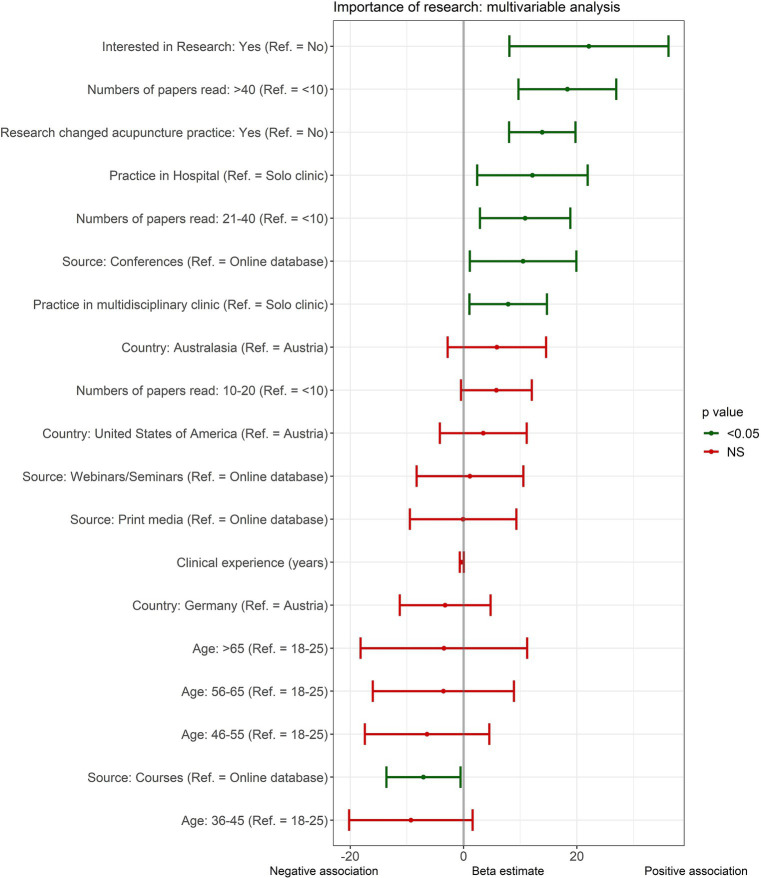
Multivariable analysis of factors associated to the importance of research in clinical practice. NS, Not significant; Ref., reference.

Based on the univariable analysis, all variables with a *p*-value less than 0.1 were included in the multivariable analysis. To build a model with best model fit, an exhaustive search for the final variable selection was performed, with the highest adjusted R-squared value. The resulting regression model had a good model fit explaining 38% of the variability (adjusted R-squared) with an overall significant regression (F[19, 241] = 9.24, *p* < 0.01). Further, the results showed that being interested in research (*ß* = 22.1, *p* < 0.01), reading more than 40 papers (*ß* = 18.2, *p* < 0.01) or 21–40 papers (*ß* = 10.9, *p* = 0.01) per year, if an acupuncture study ever changed the clinical practice (*ß* = 13.9, *p* < 0.01), practicing in a hospital (*ß* = 12.2, *p* = 0.01) or multidisciplinary clinic (*ß* = 7.9, *p* = 0.02), and attending conferences to inform the clinical practice of acupuncture (*ß* = 10.5, *p* = 0.03) was strongly associated to rating research as more important for clinical practice. On the other hand, using certified courses for CPD (*ß* = −7.1, *p* = 0.03) was negatively associated to the importance of research in clinical practice rating (Figure 4).

## 4 Discussion

Factors associated to a favorable attitude toward acupuncture research were identified in this large multinational survey among acupuncturists. While not surprising that acupuncturists rate research as more important for clinical practice when being interested in research or with the numbers of papers read per year, the most interesting finding was the independent prognostic effect of the place of practice, source of information, and if an acupuncture study ever changed the clinical practice of the respondents. However, it appears that current programs of acupuncture education commonly lack a focus on developing basic research skills including searching and interpreting adequate sources. We therefore outlined and discussed each of the identified factors below.

Overall, our identified factors could serve as basic principles for strategies that aim at promoting research literacy among acupuncturists. Previous studies identified interactive and activity-based learning as effective to improve the research literacy of healthcare personnel (3) and acupuncturists (6). Our results show a strong statistical association between the value of research and if an acupuncture study ever changed acupuncturists’ clinical practice. It is likely that positive experiences with research in clinical practice improve the interest and value of research in general. Emphasizing the practical value of acupuncture studies rather than lecturing theoretical principles of research methodology should be a core principle of future educational programs, which could be achieved by focusing on the critical evaluation and extraction of clinically useful information such as acupuncture points used and aspects of dosage regime, including treatment frequency and duration.

Further, interdisciplinary information exchange is another important factor that is associated to a higher value of research in clinical practice (3). This is especially highlighted by our results in the uni- and multivariable analysis showing conferences being positively and certified courses being negatively associated with the importance of research in clinical practice rating. Conferences are an important outlet for researchers to present, disseminate, and discuss their findings with attendees, whereas CPD courses are often held by experts in their field who approach acupuncture theory from a traditional angle of knowledge and are rarely evidence-informed in a modern sense (21). In general, future educational programs should not only incorporate more research findings to justify the use of acupuncture for certain conditions or treatment approach using the available scientific evidence, but should also facilitate interdisciplinary exchange through, for example, mandatory participation at acupuncture conferences as attendee or possibly presenter in poster sessions. For this purpose, acupuncturists can use data collected in their own clinic, accurately reflecting real-world acupuncture practice and outcomes, which they can also publish in dedicated Case Report journals, therefore simultaneously contributing toward informing future trial design and actively closing the loop of evidence-informed practice and practice-informed research (24).

Another example of how inter- and multidisciplinary environments predicted a higher research literacy are the implementation of acupuncturists in public health care facilities including hospitals and multidisciplinary clinics. Our results showed significant higher importance of research in clinical practice ratings of acupuncturists working in multidisciplinary clinics or hospitals compared to those working in their own solo clinic. Additionally, working in either of these settings were significant predictors of the importance rating in our multivariable analysis. In public healthcare facilities, essential requirements concerning evidence-informed practice are generally higher than in solo clinics, challenging acupuncturists to further educate themselves in research literacy skills (30). With the progressing implementation of acupuncture in public healthcare facilities, future educational programs should consider clinical internships in teaching hospitals as a possibility to foster multidisciplinary information exchange among acupuncture students and practitioners.

To the best of our knowledge, previous surveys assessing the attitude and role of research for evidence-informed practice among acupuncturists are rare and were on national level only, not allowing strong general conclusions or the assessment of cultural differences. Furthermore, these studies were of descriptive nature and did not use inferential statistical processes to quantify relationships (4, 6, 21, 23). However, the results of these studies complement our findings of research being underutilized as a source of information for clinical practice among acupuncturists. Practitioners mostly attend CPD courses (39.3%) to educate themselves further and value experts’ opinion as the most reliable source of information (43.1%). Using CPD courses as primary source of information was additionally unfavorably associated to valuing research as important for clinical practice. On the other hand, only 27.6% use online databases such as Pubmed or Google Scholar as primary source of information and 35.5% trust research papers the most according to our results. Dahle et al. and Graca et al. reported similar results for the incorporation of research findings in clinical practice for Norway and Australasia (39 and 27%, respectively) (6, 21). Additionally, reading more than 20 research papers per year was an independent predictor of a higher value of research for clinical practice in our sample. Another positive predictor was if acupuncturists ever participated in acupuncture research. Anderson et al. found that research literacy was positively correlated to research participation in faculty members of an oriental medicine college in the United States of America (23). However, this study was performed among members of a highly academic environment potentially explaining the positive correlation. Contrarily, our results showed a weak statistical association of the highest education level in general as well as in acupuncture to the importance of research rating. Further, adding these variables to our multivariable model did not increase the variability explained, suggesting a low relevance of the educational background for the value of research of acupuncture practitioners in clinical practice.

We did not identify any influence on the value of research of nation-level characteristics even though respondents in Germany showed significant lower importance of research ratings. This could be partially explained by the German educational system of acupuncturists, where it is not required to hold an academic degree in order to become a licensed acupuncturist. By contrast, in Austria only medical doctors are allowed to perform acupuncture, and in the United States of America and Australasia, acupuncturists need at least a Bachelor’s degree to become a licensed acupuncturist (31–35). German acupuncturists may therefore experience the least education in scientific principles, potentially explaining the low rating of the importance of research for their clinical practice. However, neither education in general, education in acupuncture, nor country of practice improved the variability explained in our multivariable analysis, suggesting once more no significant impact on the value of research. Additionally, the majority of respondents stated that the principles of TCM remain valid in the context of modern medicine and can be replicated in a research study, suggesting a positive attitude toward research in principle, but most acupuncturists seem to lack the skills to interpret and use available research findings in clinical practice, resulting in the low importance rating. Previous studies showed comparable results, confirming that the educational background had no direct influence on the research literacy, the critical assessment of scientific information, or protection against health misinformation (36).

There are certain limitations to this study including the overall low response rate to the survey. However, this is not uncommon in surveys using a convenience-sampling design and may be due to several factors, including low interest in actively participating in research (37). Additionally, some items of the survey have a moderately low response rate compared to others which needs to be considered when interpreting the results. Overall, the strengths of our study include the multi-national design allowing for more general conclusions for future educational programs and health care policy measurements aiming to improve the research literacy of acupuncturists.

In conclusion, the results provide first details about significant factors relevant for the research literacy of acupuncturists in western countries. Aiming for an interdisciplinary and interactive educational program may improve the value of research and facilitate the skills to implement evidence-informed practice among acupuncturists. Future educational programs should also be evaluated in terms of skills in research literacy and critical appraisal of research studies to enhance the quality of acupuncture training in general. Targeting the lack of evidence-informed practice will also help to further implement acupuncture in public health care systems through generally improving the acceptability by balancing the interventions effectiveness and patient safety.

## Data availability statement

The raw data supporting the conclusions of this article will be made available by the authors, without undue reservation.

## Ethics statement

The studies involving humans were approved by Ethics Committee of the Medical University of Graz, Austria. The studies were conducted in accordance with the local legislation and institutional requirements. The participants provided their written informed consent to participate in this study.

## Author contributions

MH: Conceptualization, Data curation, Formal analysis, Investigation, Methodology, Project administration, Software, Validation, Visualization, Writing – original draft, Writing – review & editing. SG: Conceptualization, Formal analysis, Investigation, Methodology, Project administration, Resources, Supervision, Validation, Writing – original draft, Writing – review & editing. SB: Formal analysis, Project administration, Supervision, Writing – review & editing. GH: Formal analysis, Supervision, Validation, Writing – review & editing. MA: Methodology, Supervision, Validation, Writing – review & editing. MP: Methodology, Resources, Supervision, Validation, Writing – review & editing.
